# Frozen section utilization to omit systematic biopsy in diagnosing high risk prostate cancer

**DOI:** 10.1038/s41598-022-18186-9

**Published:** 2022-08-24

**Authors:** Jong Hyun Tae, Hyun Jung Jin, Tae Il Noh, Ji Sung Shim, Seok Ho Kang, Jun Cheon, Jeong Gu Lee, Sung Gu Kang

**Affiliations:** 1 Department of Urology, Korea University Anam Hospital, Korea University Medical Center, Korea University School of Medicine, 73, Inchon-ro, Seongbuk-gu, Seoul, 02841 Korea; 2 Department of Urology, Chung-Ang University Hospital, Chung-Ang University College of Medicine, Seoul, Korea

**Keywords:** Surgical oncology, Prostate cancer, Cancer screening

## Abstract

The current guidelines for targeted prostate biopsy recommend an additional systematic biopsy regardless of clinical risk assessment. To evaluate frozen section biopsy utilization in targeted prostate biopsy to omit systematic biopsies in cases of positive frozen section results of patients with clinical features suggestive of high-risk prostate cancer. In this prospective, single-center study, we enrolled patients with a Prostate Imaging-Reporting and Data System (PI-RADS) 5 lesion on magnetic resonance imaging (MRI) with clinical evidence suggestive of high-risk prostate cancer (either an extracapsular extension or prostate-specific antigen level > 20 ng/ml). All patients underwent 2–4 core targeted biopsies utilizing frozen section biopsy with immediate results, allowing patients with a positive result to omit a systematic biopsy. In case of a negative result, additional systematic biopsies were performed. The primary endpoint was the detection rate of targeted biopsy. Patient demographics, clinical variables were analyzed using SPSS version 20. Sixty-six patients were enrolled in this study. Among them, 63 patients were diagnosed with cancer without the need for an additional systematic biopsy. Three patients were non-diagnostic with target biopsy alone. Hence an additional systematic biopsy was performed. Two of these patients were diagnosed with prostate cancer and one tested negative for cancer. In this report we looked into the necessity of taking a routine systematic biopsy in patients with high risk features of prostate cancer. We found that utilizing frozen section biopsy for targeted biopsy reduces unneccessary systematic biopsy in 97% of cases and still provides a means for systematic biopsy when targeted biopsy alone fails to make the diagnosis.

## Introduction

Multiparametric magnetic resonance imaging (mpMRI) is highly sensitive for detecting prostate cancer^1^. There is compelling evidence regarding the role of targeted prostate biopsy using mpMRI in guiding tissue sampling and further improving the detection rate of prostate cancer^2^. This has been demonstrated in biopsy-naïve men^3^, men with prior negative biopsy results^4,5^ and those considering active surveillance^6,7^. Additionally, magnetic resonance imaging (MRI)–ultrasonography (US) fusion-targeted biopsy techniques have been shown to improve the diagnostic yield of clinically significant prostate cancer (csPCa) while lowering the detection of clinically insignificant prostate cancer^2,8^. However, random multiple systematic biopsies combined with targeted biopsy cannot be replaced by targeted biopsies alone because 60–90% of cases show multifocality^9^, and csPCa is missed on mpMRI in up to 16–28% of the cases^10,11^. Furthermore, the combination of targeted and systematic biopsies resulted in detecting more csPCa cases than either method alone^11^. Therefore, the current literature advocates combining targeted biopsies with random multiple systematic biopsies.

In a specific subset of groups with Prostate Imaging-Reporting and Data System (PI-RADS) 5 lesions on MRI, Mehralivand et al. reported a cancer detection rate of 86.9% with targeted biopsies^12^. At our institution, the detection rate of prostate cancer for PI-RADS 5 lesions was previously reported to be 89.4%^13^. Despite the high cancer detection rate of prostate cancer for PI-RADS 5 lesions with targeted biopsy, a systematic biopsy is routinely performed because of the potential risk of missing csPCa. Though patients with clinical evidence suggestive of high-risk prostate cancer (either an extracapsular extension or prostate-specific antigen [PSA] level > 20 ng/ml) have csPCa regardless of the Gleason score (GS) if diagnosed with prostate cancer, a systematic biopsy cannot be performed according to current guidelines. It is speculated that a routine systematic biopsy can be safely omitted for patients with clinical features of high-risk prostate cancer and a PI-RADS 5 lesion on MRI if the results of the target biopsy could be immediately identified. In cases where cancer is identified, a systematic biopsy can be omitted. In contrast, in case of a negative target biopsy result, performing an additional systematic biopsy would help in adhering to the current guidelines. Therefore, in this study, we used a protocol that utilizes frozen section biopsies to omit systematic biopsies in cases of positive results for cancer or additionally perform systematic biopsies in cases of negative results to mitigate the potential risk of missing prostate cancer. This study aimed to investigate the cancer detection rate for PI-RADS 5 lesions in high-risk patients (with an extracapsular extension or high PSA level > 20 ng/ml) and prospectively verify our protocol and effectiveness of utilizing frozen sections to omit unnecessary systematic biopsies in individuals with high-risk prostate cancer.

## Materials and methods

### Study population

In this prospective study, patients from March 2018 to August 2020 with an elevated PSA (> 4.0 ng/mL) underwent biparametric MRI. The inclusion criteria for enrollment was a PI-RADS 5 region of interest (ROI) on MRI in addition to one of either of the following: (1) PSA level > 20 ng/ml or (2) suspicion for extracapsular extension on MRI. Thus, patients with high suspicion for locally advanced PCa or metastatic disease who needed only a histological diagnosis were enrolled to this study. Patients that required a systematic biopsy for accurate risk assessment were excluded from the study.

### Imaging work-up

All men with elevated PSA (4.0 ng/mL) underwent non-contrast-enhanced biparametric MRI. Bi-parametric MRI was performed using a 3.0-T scanner (Siemens Medical System, Erlangen, Germany) without the dynamic contrast-enhanced imaging sequence from mpMRI. ROIs on bpMRI were marked by three dedicated uro-radiologists based on the Prostate Imaging- Reporting and Data System (PI-RADS), version 2.0. ROIs were set in areas with PI-RADS 5 on bpMRI and used as targeted regions. An MRI–US fusion device was used (BioJet, Geoscan, Lakewood Ranch, Fla), which had previously been approved for clinical use by the U.S. Food and Drug Administration.

### Study protocol and procedure

#### Procedure

MRI–US fusion-targeted transperineal prostate biopsies were performed under monitored anesthesia care (MAC), which uses a slow injection of 0.5 mg/kg of propfopol or midazolam for initiation of sedation and 25–75 mcg/kg/min IV during the first 10–15 min and subsequently decreased to 25 to 50 mcg/kg/min for maintenance with bispectral index(BIS) monitoring to range between 60 and 80. In the elderly, the dose is reduced to approximately 60–80% of the usual dose. For pain control, remifentanil or fentanyl was used additionally. The procedure for MRI-US fusion-targeted transperineal prostate biopsy was previously described^13^.

#### Prostate biopsy protocol

The protocol used focuses on utilizing frozen section biopsy to achieve immediate results to render patients free from additional systematic biopsies.

The protocol was as follows: all patients had 2–4 cores of frozen section specimens sent to the pathologist. Based on the immediate results of the frozen section biopsy, systematic biopsy was omitted when the results were positive for cancer. However, when the results were negative for cancer, a 16-core systematic biopsy was performed.

### Primary endpoint

The primary endpoint of this study was the detection rate of targeted biopsy for prostate cancer in high-risk patients with a PI-RADS 5 lesion on MRI using frozen section biopsy.

### Statistical analysis

Descriptive analysis for continuous variables are expressed as median (interquartile range [IQR]), mean (standard deviation) or as a number (percentages). Categorical variables are reported as numbers and percentages. All analyses were performed using the Statistical Package for the Social Sciences(SPSS) version 20.0(IBM Corp., Armonk, NY, USA).

### Ethical statement

This study was approved by the Institutional Review Board of Korea University Hospital (No. 2018AN0339). Informed consent was obtained from all participants. All methods used in this study complied with the relevant guidelines and regulations and in accordance with the Declaration of Helsinki.

## Results

The demographic and clinical characteristics of patients are presented in Table 1. A total of 66 patients were enrolled in this study. The median age was 73.4 years (interquartile range [IQR], 66.0–80.0 years), and the median PSA level was 60.0 ng/ml (IQR, 14.0–212.5 ng/ml). The median prostate volume was 35.2 cm^3^ (IQR, 28.4–55.2 cm^3^). The mean tumor size on MRI (length of the longest axis) was 2.4 cm (IQR, 1.7–3.6 cm). A digital rectal examination revealed that 50.0% of the patients (n = 33) had positive findings of a palpable nodule. A hypoechoic lesion was detected in 74.2% (n = 49) of the patients. Of all patients, 12.1% (n = 8) had a history of negative biopsy results. In total, 15.9%, 47.7%, and 36.4% of the cases were clinically localized (T2N0), locally advanced (T3-4N0), and distant metastatic diseases (TxN1 or M1), respectively.

**Table 1:** Patient characteristics.

	Median/number	IQR
Patients (n)	66	
Median age, yr (IQR)	73.4	(66.0–80.0)
Median PSA, ng/ml (IQR)	60.0	(14.0–212.5)
Median PV, cm^3^ (IQR)	35.2	(28.4–55.2)
MRI tumor size (length, cm)	2.4	(1.7–3.6)
Positive DRE results, n (%)	33 (50.0)	
Positive hypoechoic lesion, n (%)	49 (74.2)	
Prior negative biopsy results, n (%)	8 (12.1)	
**Clinical stage, n (%)**		
Clinically localized, T2N0	12 (18.2)	
Locally advanced, T3-4N0	25 (37.9)	
Distant metastasis, TxN1 or M1	29 (43.9)	

IQR, interquartile range; PSA, prostate-specific antigen; PV, prostate volume; MRI, magnetic resonance imaging; DRE, digital rectal examination.

**Figure 1:**
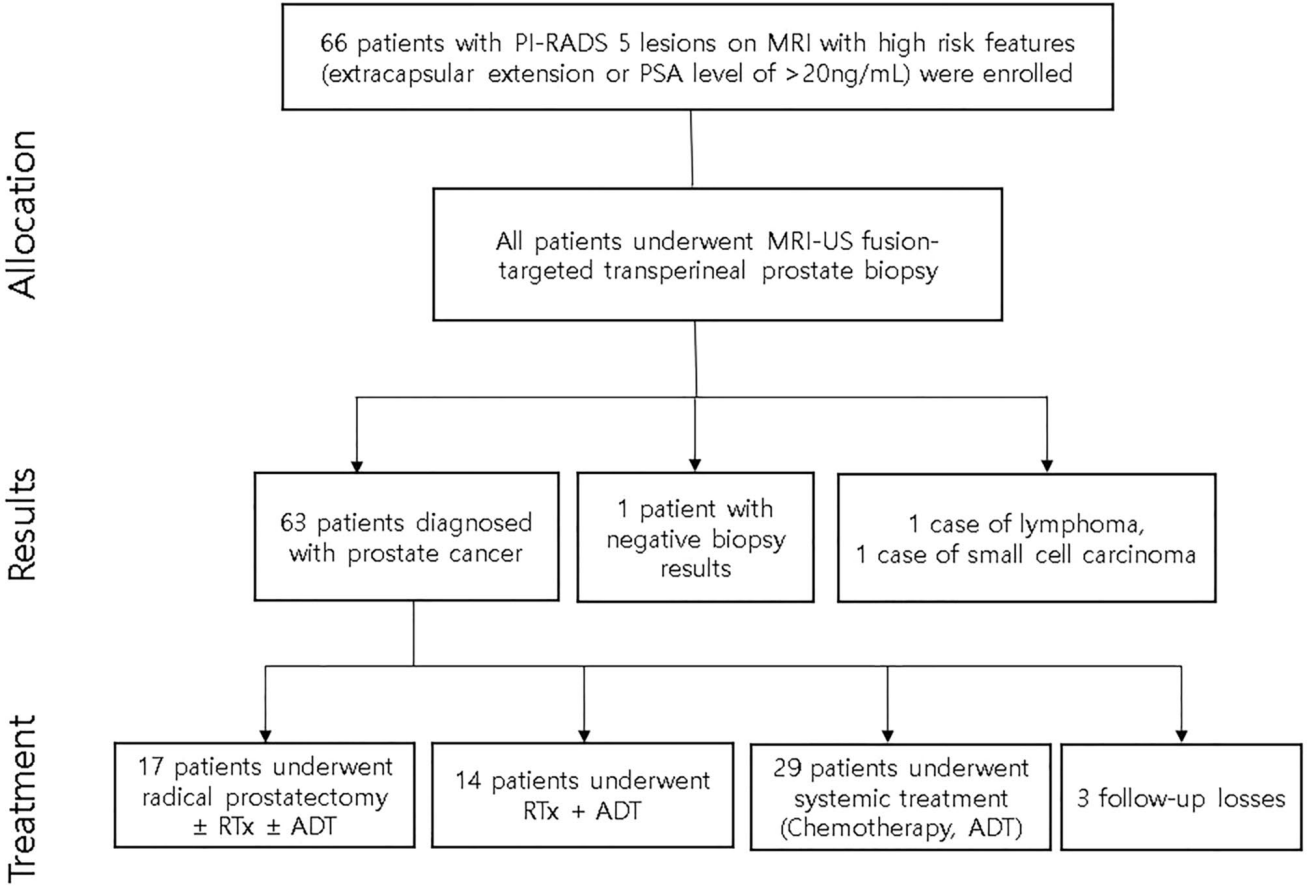
Flowchart of the study from patient selection to treatment. PI-RADS, Prostate Imaging-Reporting and Data System; MRI, magnetic resonance imaging; PSA, prostate-specific antigen; US, ultrasonography; RTx, radiation therapy; ADT, androgen deprivation therapy. Created by PowerPoint 2019. Flowchart of the study from patient selection to treatment © 2022 by Jong Hyun Tae is licensed under Attribution 4.0 International. To view a copy of this license, visit http://creativecommons.org/licenses/by/4.0/.

The targeted biopsy results are summarized in Table 2. Among the 66 patients, 65 were diagnosed with cancer, 63 with adenocarcinoma, 1 with lymphoma, and 1 with small-cell carcinoma (Fig. 1). The average number of cores obtained from the biopsies was 2.9 cores. A total of 2.2 frozen cores were taken on average. The histopathological results were adenocarcinoma in 95.5% (n = 63) of the cases and lymphoma and small-cell carcinoma in 1.5% (n = 1), each.

**Table 2:** Results of MRI–US fusion-targeted prostate biopsy with utilization of frozen section biopsy in high-risk prostate cancer.

	Mean/number
Total biopsy cores, mean ± SD	2.9 ± 1.4
Frozen section biopsy cores	2.2 ± 0.7
Total Procedure time, mean ± SD (min)	25.3 ± 5.7
**Pathology, n (%)**	
Adenocarcinoma	63 (95.5)
Lymphoma	1 (1.5)
Small-cell carcinoma	1 (1.5)
Negative for cancer	1 (1.5)
**Detection rate, N**_**positive**_**/N**_**total**_** (%)**	
Frozen section biopsy cancer detection rate	63/65 (97.0)
Positive frozen section biopsy cores/Total frozen section biopsy cores	126/146 (86.3)

MRI, magnetic resonance imaging; US, ultrasonography; SD, standard deviation.

The primary endpoint of this study was the targeted biopsy cancer detection rate using frozen sections from high-risk patients with a PI-RADS 5 ROI. The cancer detection rate was 97.0% (n = 63/65). These patients were diagnosed with cancer using targeted biopsy only and did not require additional systematic biopsies. In three cases, the results of the targeted biopsy were negative, and these patients underwent an additional systematic biopsy in which two cases of prostate cancer were further identified. Excluding the results of the one case that tested negative for cancer, the positivity rate of the frozen section biopsy results was 86.3% (126 cores positive/146 total cores obtained).

Formalin-fixed paraffin-embedded pathology reports for accurate GSs were obtained from frozen sections and reported by a pathologist such as it would be in a regular biopsy scheme. There were eight cases classified as grade group 1 in the initial targeted biopsy in which five patients received radical prostatectomy, two patients received chemotherapy with androgen deprivation therapy (ADT), and one patient received hormonal therapy. Among the five patients who were classified as grade group 1 that underwent radical prostatectomy, four patients were upgraded to grade group 2 and one patient to grade group 3 in the final pathology report. All five patients had a definite extracapsular extension on MRI, suggesting csPCa despite a GS of 6 on biopsy; their final pathological results were consistent with the clinical findings of T3aN0 in four cases and pT3bN0 in one case.

Among the 66 patients, there were three follow-up losses: one patient had small-cell carcinoma, one had lymphoma, and one had a negative biopsy result. The treatments received by patients diagnosed with adenocarcinoma (n = 60) using frozen section biopsies are summarized in Tables 3 and 4. Seventeen of the 60 patients underwent radical prostatectomy. Forty-three patients did not undergo surgery for advanced prostate cancer or a high-risk comorbid status; among them, 14 (23.3%), 21 (35.0%), and 8 (13.3%) patients were treated with radiation therapy plus ADT, hormone therapy alone, or hormone therapy in combination with chemotherapy, respectively.

**Table 3:** Treatment course of the patients (n = 60).

	Mean/number
Type of treatment, n (%)	60*
Radical prostatectomy + PLND	11 (18.3)
Radical prostatectomy + ADT + RTx	6 (10.0)
RTx + ADT	14 (23.3)
Hormonal therapy	21 (35.0)
CTx + ADT	8 (13.3)

PLND, pelvic lymph node dissection; ADT, androgen deprivation therapy; RTx, radiation therapy; CTx, chemotherapy (docetaxel).

*Among the 66 patients, there were three follow-up losses, one case of small-cell carcinoma, one case of lymphoma, and one case of a negative biopsy result.

**Table 4:** Treatments received according to Gleason’s score.

Gleason’s score	Treatment (n = 60)^a^					
	RP	RP + RTx/ADT	RP + ADT	ADT	RTx/ADT	CTx/ADT
6	4	1	0	1	0	2
7	3	1	1	5	2	0
8	4	2	1	12	12	4
9	0	0	0	1	0	1
10	0	0	0	2	0	1

RP, radical prostatectomy; ADT, androgen deprivation therapy; RTx, radiation therapy; CTx, chemotherapy (docetaxel); ^a^Only the patients diagnosed with adenocarcinoma are shown.

No postoperative complications, such as pain, urinary retention, urinary tract infection, or hematuria, were noted postoperatively in all 66 patients.

## Discussion

The high sensitivity of MRI has led to its incorporation into the diagnostic pathway as an initial test prior to biopsy. A systematic review on mpMRI detection of prostate cancer reported a sensitivity of 93%, a negative predictive value of 89%, and a specificity of 41%^1^. With the introduction of MRI, prostate biopsy techniques have evolved to further improve the diagnostic uncertainty of previous random biopsies. Targeted prostate biopsy utilizing MRI for guiding tissue sampling has been reported to improve the detection rate of csPCa while lowering the detection of clinically insignificant prostate cancer^2^. However, targeted biopsy alone is not recommended in most reports owing to the undetected csPCa on mpMRI in up to 16–28% of the cases and the multifocality of prostate cancer, which is known to be present in 60–90% of cases^9–11^. Therefore, current guidelines suggest that targeted prostate biopsies should be performed in combination with a systematic biopsy for a more accurate risk stratification that could further impact treatment decisions. However, the high detection rate of targeted biopsy for PI-RADS 5 lesions suggest that the detection rate of a targeted biopsy could be further increased and potentially warrant the omission of systematic biopsies in more specifically selected patients. Therefore, patients with a PIRADS-5 lesion on MRI with additional high-risk features (with an extracapsular extension or PSA level > 20 ng/mL) may benefit from a frozen section biopsy because these subgroup of patients need only a histologic diagnosis without further risk assessment with a systematic biopsy. Frozen section biopsy provides immediate results to omit systematic biopsy in cases of a positive result for cancer; however, a 16-core systematic biopsy can be performed in cases of a negative result to mitigate the potential risk of missing prostate cancer.

The cancer detection rate for high-risk patients with frozen section-targeted biopsy alone was 97.0% (63/65 patients). There were three cases where an additional systematic biopsy was performed owing to the negative results of a targeted biopsy using frozen sections. In one case, an additional systematic biopsy was performed, and the pathology was reported to be a prostate abscess. If we were to have performed a routine systematic biopsy in combination with targeted biopsy for these high-risk patients with PI-RADS 5 lesions, 95.5% (63/66) of these patients would have undergone an additional systematic biopsy without any benefit.

Regarding treatment course, 31 (51.7%) patients underwent whole-gland treatment initially with radical prostatectomy or radiation therapy. Twenty-nine (48.3%) patients underwent systemic therapy with hormonal therapy alone or in combination with chemotherapy. An additional systematic biopsy would not have deviated the course of treatment for these patients.

The most commonly reported minor complications related to transrectal biopsies were hematospermia (36.3%), hematuria (14.5%), and rectal bleeding (2.3%). Rectal bleeding requiring intervention was reported in 0.6% of the patients. Other reported complications include urinary tract infection (0.8%) and urinary retention (0.2%)^14^. Additionally, the risks of antimicrobial resistance and urosepsis have been reported to increase with the number of cores sampled^15^. Though the biopsy technique adopted in this study utilizes a transperineal approach, it is speculated that frozen sections utilized in a transrectal approach for target biopsy may provide similar benefits of a significant reduction in the number of biopsy cores. No complications associated with the procedures were noted in any of the 66 patients in this study.

The major advantage of incorporating a frozen section biopsy is that the total biopsy cores obtained can be significantly reduced by omitting a systematic biopsy without compromising the risk of missing diagnoses of prostate cancer. Reducing the number of cores sampled may lower the risk of antimicrobial resistance and urosepsis, as they have been reported to increase with the number of cores sampled^15^. Furthermore, the time from suspicion to diagnosis and eventually to treatment is shortened, and the anxiety experienced by patients in these intervals is lowered. However, when an accurate risk assessment is required, a systematic biopsy should be added to targeted biopsy cores to improve the local staging in order to plan the optimal therapeutic strategy^16^.

The procedure presented in this study has limitations. Obtaining frozen section biopsy results requires a pathologist to be readily able to assess the specimen, which may not be possible in local practices. However, by omitting a routine systematic biopsy, the total number of biopsy cores that need to be reviewed by the pathologist can be significantly reduced, leading to a decrease in the burden on pathologists as well. In addition, time for a pathologic report for frozen section can be time consuming and may exceed performing a systematic biopsy. The total operation time consumed in this study was a mean 25.3 ± 5.7 min which is similar to MRI–US fusion-targeted transperineal prostate biopsies with systematic biopsies that are performed in our institute. However, a reduction in biopsy cores may enable this method to be performed in an out-patient setting under local anesthesia where the patient can wait in the waiting room until the pathology result is reported. Another limitation is that MRI must be performed preoperatively to perform an MRI fusion biopsy, which can be expensive in certain countries. However, owing to the national health insurance policy in Korea, the patients are not economically burdened. Lastly, the number of cases included in this study was small; a larger trial is required to make any definitive conclusions.

## Conclusions

In selected patients with a PI-RADS 5 lesion and clinical features of high-risk prostate cancer, a targeted frozen section biopsy may be clinically utilized to rapidly confirm the existence of carcinoma and render the patient free of additional systematic biopsies as it will not deviate from the treatment course for the patient.
