# Author Correction: Frozen section utilization to omit systematic biopsy in diagnosing high risk prostate cancer

**DOI:** 10.1038/s41598-022-22616-z

**Published:** 2022-11-01

**Authors:** Jong Hyun Tae, Hyun Jung Jin, Tae Il Noh, Ji Sung Shim, Seok Ho Kang, Jun Cheon, Jeong Gu Lee, Sung Gu Kang

**Affiliations:** 1 Department of Urology, Korea University Anam Hospital, Korea University Medical Center, Korea University School of Medicine, 73, Seoul, 02841 Korea; 2 Department of Urology, Chung-Ang University Hospital, Chung-Ang University College of Medicine, Seoul, Korea

Correction to: *Scientific reports* 10.1038/s41598-022-18186-9, published online 24 August 2022

The original version of this Article omitted an affiliation for Jong Hyun Tae. The correct affiliations are listed below.

Department of Urology, Korea University Anam Hospital, Korea University Medical Center, Korea University School of Medicine, 73, Inchon‑ro, Seongbuk‑gu, Seoul 02841, Korea

Department of Urology, Chung-Ang University Hospital, Chung-Ang University College of Medicine, Seoul, Korea

The original Article has been corrected.

